# Th1-Induced CD106 Expression Mediates Leukocytes Adhesion on Synovial Fibroblasts from Juvenile Idiopathic Arthritis Patients

**DOI:** 10.1371/journal.pone.0154422

**Published:** 2016-04-28

**Authors:** Laura Maggi, Francesca Margheri, Cristina Luciani, Manuela Capone, Maria Caterina Rossi, Anastasia Chillà, Veronica Santarlasci, Alessio Mazzoni, Rolando Cimaz, Francesco Liotta, Enrico Maggi, Lorenzo Cosmi, Mario Del Rosso, Francesco Annunziato

**Affiliations:** 1 Department of Experimental and Clinical Medicine, University of Florence, Florence, Italy; 2 Regenerative Medicine Unit, Careggi University Hospital, Florence, Italy; 3 Department of Experimental and Clinical Biomedical Sciences, University of Florence, Florence, Italy; 4 Department of Clinical and Experimental Medicine and Centre for Biomolecular Studies Supporting Human Health, Second University of Naples, Naples, Italy; 5 Department of Paediatrics, Rheumatology Unit, Anna Meyer Children’s Hospital and University of Florence, Florence, Italy; Queen Mary University of London, UNITED KINGDOM

## Abstract

This study tested the hypothesis that subsets of human T helper cells can orchestrate leukocyte adhesion to synovial fibroblasts (SFbs), thus regulating the retention of leukocytes in the joints of juvenile idiopathic arthritis (JIA) patients. Several cell types, such as monocytes/macrophages, granulocytes, T and B lymphocytes, SFbs and osteoclasts participate in joint tissue damage JIA. Among T cells, an enrichment of classic and non-classic Th1 subsets, has been found in JIA synovial fluid (SF), compared to peripheral blood (PB). Moreover, it has been shown that IL-12 in the SF of inflamed joints mediates the shift of Th17 lymphocytes towards the non-classic Th1 subset. Culture supernatants of Th17, classic and non-classic Th1 clones, have been tested for their ability to stimulate proliferation, and to induce expression of adhesion molecules on SFbs, obtained from healthy donors. Culture supernatants of both classic and non-classic Th1, but not of Th17, clones, were able to induce CD106 (VCAM-1) up-regulation on SFbs. This effect, mediated by tumor necrosis factor (TNF)-α, was crucial for the adhesion of circulating leukocytes on SFbs. Finally, we found that SFbs derived from SF of JIA patients expressed higher levels of CD106 than those from healthy donors, resembling the phenotype of SFbs activated in vitro with Th1-clones supernatants. On the basis of these findings, we conclude that classic and non-classic Th1 cells induce CD106 expression on SFbs through TNF-α, an effect that could play a role in leukocytes retention in inflamed joints.

## Introduction

Inflammatory responses play a key role in host defense from foreign agents but can be also responsible of tissue damage, for example in autoimmune diseases. The function of T cells is to recognize specific “non-self” antigens and to generate specific responses tailored to eliminate the pathogen. CD4+ T cells can be functionally subdivided into two main subsets: effector cells, which provide protection against exogenous offending agents, and regulatory T (Treg) cells whose function is to avoid autoimmune reactions and to stop the effector response against exogenous antigens, when the response itself becomes dangerous for the host [1]. Human effector CD4+ T lymphocytes can be additionally classified into subpopulations based mainly on their immunological functions. Th1 cells express the transcription factor T-bet, secrete interferon (IFN)-γ, and protect the host from intracellular infections. Th2 cells express GATA-3, secrete interleukin (IL)-4, IL-5, IL-9 and IL-13 and are involved in protection from helminths [2, 3]. Th17 cells defend the body from extracellular bacterial and fungal infections [4–7], express the transcription factor RORC [8], the IL-23 receptor (IL-23R), the chemokine receptor CCR6 [9, 10], and the lectin receptor CD161 [11]. Beyond their protective role in the clearance of extracellular pathogens, Th lymphocytes have been described to play a role in the pathogenesis of several autoimmune and inflammatory diseases, such as multiple sclerosis, inflammatory bowel disease (IBD), psoriasis, rheumatoid arthritis (RA), and JIA [1, 12], but also atopic disorders. JIA is the most common form of persistent arthritis in children. Even if the cause of disease is still poorly understood [13], adaptive immune responses are certainly involved in its pathogenesis, as indicated by the presence of T and B lymphocytes infiltrating the synovial membrane of inflamed joints [14]. T-cell infiltrates predominantly consist of CD4+ Th1 cells which have been thought to have a central role in the pathogenesis of the disease [15, 16]. Recently, we reported an accumulation of CD4+CD161+ cells, belonging to either the nonclassic Th1 or the Th17/Th1 subset, in the inflamed joints of JIA patients, and we showed that their proportions in synovial fluid (SF) positively correlated with parameters of disease activity [17]. Accordingly, the shifting of CD4+CD161+ cells from the Th17 to the non-classic Th1 phenotype has been shown to occur in the SF of JIA children [17, 18]. The CD4+ T cells orchestrate the chronic inflammation in both RA and JIA by acting, through the production of cytokines, on multiple cell types found in inflamed joints. Among these, SFbs are certainly the most important tissue resident cell population in the synovium. Extensive studies in adult RA have shown the existence of SFbs that produce cytokines and matrix-degrading enzymes, thus playing a crucial role in cartilage destruction and inflammation [19].

In this study we examined the ability of different subset of T helper cells to activate SFbs to produce/express molecules involved in leukocytes retention in the inflamed synovia.

## Materials and Methods

### Patients and samples

Synovial fibroblasts were derived from 7 JIA patients (age mean: 9 years, range: 9–15 years; none was at baseline, 4 of them were treated with NSAD and 3 with MTX) and 6 healthy controls (age mean: 8 years, range: 5–10 years), after informed written consent and with the approval of Ethics Committee of Anna Meyer Children Hospital, Florence, Italy. For patients under the age of 18 years, written consent was obtained by parents or guardians. All the procedures of this study have been conducted according to the principles expressed in the Declaration of Helsinki. Synovial fluid samples were obtained during arthrocentesis from the knee joint aspirates of seven oligoarticular JIA patients, while synovial tissue was obtained from the knee joints of four healthy subjects undergoing orthopaedic surgery for knee traumatic events. These donors didn’t suffer from any chronic inflammatory diseases and didn’t take any drug, for this reason they were called healthy.

### Synovial fibroblasts isolation and culture

Synovial tissue was minced into small pieces and plated in culture dishes with FBM-2 (Fibroblast Growth Medium, Microtech, Napoli, Italy) supplemented with 10% FBS for expansion, as previously described [20, 21]. JIA SFbs were obtained from synovial fluid of JIA patients. Briefly, synovial fluid was collected, centrifuged, washed twice in PBS (Phosphate Buffered Saline, Microtech, Napoli, Italy) and cells were plated in FBM-2 supplemented with 10% FBS (Fetal Bovine Serum). The day after plating, non-adherent cells were removed. The cell monolayers were used within the 7th passage in culture. The cells were considered type fibroblast-like synovial cells if positive on staining with anti-CD105, anti-CD29 (Ancell, Bayport, MN, USA), anti-CD73 and anti-CD90 (BD Biosciences), and negative for anti-CD31, anti-CD45, anti-CD34, anti-CD14, anti-CD16 (BD Bioscience) and if they had a spindle-shaped, fibroblast-like morphology.

### T cells isolation and characterization

CD4+ T cells, derived from PB mononuclear cells (MNCs) of four healthy donors by using the CD4 isolation kit II (Miltenyi Biotec, Bergisch Gladbach), were further divided into CD161+ and CD161- T-cell fraction by staining with an anti-CD161-PE mAb, followed by incubation with an anti-PE microbead mAb (Miltenyi Biotec). The CD4+CD161+ and CD4+CD161- cell subsets were then cultured under limiting dilution (0.3 cell/well) in presence of 10^5^ irradiated (9000 rad) allogeneic PBMCs as feeder cells, 1% PHA (vol/vol), and 50 U/mL rIL-2 (Proleukin, Prometheus, Inc., San Diego, USA), in order to obtain T-cell clones. Recovered CD4+ T-cell clones were classified on the basis of their ability to produce IFN-γ and/or IL-17 and to express surface marker CD161, as previously described [22]. Briefly, T cells were polyclonally stimulated with PMA plus ionomycin for 6 hours the last 4 in presence of Brefeldin A (BFA), fixed in formaldehyde and then analysed for intracellular cytokines production on a BDLSR II flow cytometry (BD Biosciences). Selected T cell clones with the following phenotypes: Th17 (CD161+IL-17+IFN-γ-), non-classic and classic Th1 (CD161+IL-17-IFN-γ+ and CD161-IL-17-IFN-γ+, respectively), were polyclonally stimulated with anti-CD3 plus anti-CD28 mAbs (human T Cell Activation/Expansion Kit, Miltenyi Biotec), for 72 hours to obtain culture conditioned supernatants, that were stored at -30°C. IFN-γ, TNF-α and IL-17 cytokine levels in T cell clones derived supernatants were evaluated by CBA flex set assay following the manufacturer instruction (BD Bioscience).

### Cell viability measurement using the WST-1 assay

The viability of normal SFbs cultured under various conditions was determined by a cell proliferation assay using the Water-Soluble-Tetrazolium-salt (WST-1) reagent (Roche Italia, Milano, Italy). WST-1 is a water-soluble sulfonated tetrazolium salt that is cleaved by cellular succinate-dehydrogenases in living cells, yielding dark blue formazan. Damaged or dead cells exhibit reduced or no dehydrogenase activity. Briefly, the cells were seeded in 96-well plates at a final density of 5 × 10^3^ cells/well, and cultured with unstimulated or CD3/CD28-stimulated culture supernatants from Th1 (classic and non-classic) or Th17 clones, or with recombinant TNF-α (10ng/ml), IFN-γ (5 ng/ml) and their combination (R&D Systems). Then 10 μl of WST-1 solution was added to each well, and the cells were incubated for 2h at 37°C. The optical density was measured using an absorbance microplate reader (Bio-Rad, Milano, Italy) at a wavelength of 450 nm. Percentage of cell viability was calculated based on the absorbance measured relative to that of the untreated control cells maintained in culture medium alone.

### Flow cytometry analysis

Healthy subjects-derived SFbs were grown in T25 flasks and incubated for 48h in medium alone or with unstimulated or CD3/CD28-stimulated culture supernatants from Th1 (classic and non-classic) or Th17 clones (or with recombinant TNF-α (10 ng/ml), IFN-γ (5 ng/ml) and their combination. When indicated, SFbs were cultured for 48h in medium alone or with culture supernatants from Th1 cells (classic and non-classic) or with TNF-α plus IFN-γ in presence or absence of the TNF-α blocker Etanercept (ETN) 10 μg/ml (Pfizer). Then SFbs were recovered and stained with fluorochrome-conjugated mAbs anti-CD29, -CD73, CD90, -CD105, -CD106 for 20 minutes on ice in dark. After washing in PBS plus Bovine Serum albumin 0.5% (BSA), cells were analysed by flow cytometry BD LSR II with DivaSoftware (BD Biosciences). The same flow cytometric analysis were performed also on SFbs derived from SF of JIA patients.

MNCs, derived from PB and SF of JIA patients after centrifugation on Ficoll–Hypaque gradient, were policlonally stimulated with PMA plus ionomycin for 6 hours the last 4 in presence of BFA, fixed in formaldehyde and then analysed for intracellular cytokine production in association with surface markers, on a BDLSR II flow cytometry (BD Biosciences).

### Quantitative Real-Time PCR analysis

Total RNA was prepared using Nucleospin RNA II (Macherey-Nagel, Carlo Erba Reagents, Milano, Italy), agarose gel checked for integrity, and reverse transcribed with GoScript system (Promega, Milano, Italy) using random primers according to manufacturer’s instructions. CD106 expression in normal SFbs under various conditions was determined by a quantitative Real-Time (RT)–PCR with an Applied Biosystem 7500 Fast Real Time PCR System (Applied Biosystems, Milano, Italy) and determined by the comparative Ct method using 18S ribosomal RNA as the normalization gene. Amplification was performed with the default PCR setting: 40 cycles of 95°C for 15 seconds and of 60°C for 60 seconds using SYBR Green–based detection (GoTaq qPCR Master Mix; Promega). Primers (IDT, Tema Ricerca, Bologna, Italy) used for RT-PCR were as follows:

-18S-rRNA: sense, 5′-CCAGTAAGTGCGGGTCATAAG-3′

   antisense, 5′-GCCTCACATAACCATCCAATC-3′;

-CD106: sense, 5′-GGGACCACATCTACGCTGACA-3′

   antisense, CCTGCTCTGCATCCTCCAGAAA-3′

TNFR1β and IFNγR2 expression was determined by Taq-Man RT-PCR, as described elsewhere (22). Primers and probes used were purchased from Applied Biosystems. Results were defined by comparative Ct method using GAPDH as the normalization gene.

All data are presented in the graphs as fold change respect to the control (untreated condition).

### Leukocyte isolation and adhesion assay

Peripheral blood leukocytes were isolated from whole venous blood of healthy individuals after hypotonic lysis of red cells. Leukocytes were labelled with 10μM carboxyflorescein succinimydil ester (CFSE, Molecular Probe, Eugene, OR.) at 37°C for 30’ in PBS and subsequently washed by centrifugation. Normal SFbs were grown on glass coverslips and incubated for 48h with Th1 (classic and non-classic) not stimulated or stimulated culture supernatants or with TNF-α and IFN-γ. A set of experiments was performed with JIA SFbs. All condition were performed in duplicate. Confluent treated normal SFbs or JIA SFbs were incubated with CFSE-labelled leucocytes (1 x 10^6^ cells) at 37°C for 2h in presence or absence of anti-CD106 mAb clone 1.G11B1 or of isotype control mAb (Southern Biotech, Birmingham, AL, USA). Non-adherent leukocytes were removed and gently washed with PBS. In one slide of each condition, the number of adherent leukocytes was estimated by counting CFSE-labelled cells in five randomly chosen fields per well at 100x using a fluorescent microscope. In the duplicate slide of the same condition all cells were recovered and leukocytes population were evaluated by flow cytometry for the expression of CD15, CD14, CD3 and CD19.

### Immunofluorescence analysis

Immunofluorescence was performed as previously described [23]. SFbs were grown on coverslips in their culture conditions. Once at semiconfluence, cells were treated with Th1 (classic and non-classic) stimulated culture supernatants or with TNF-α and IFN-γ. A set of experiments was performed with JIA SFbs. 48h after treatment, leucocytes CFSE-labelled were added to SFbs cells monolayers and incubated in presence or absence of anti-CD106 antibody. After two hours the slides were washed with PBS to eliminate non-adherent cells and were observed on fluorescent microscopy, as described above, to count adherent cells. Then the slides were fixed and TRITC-labelled phalloidin (Sigma, Milano, Italy) was applied to the cells to visualize cell morphology and the leucocyte adhesion on SFbs surface. Nuclei were stained with the DAPI (10μg/ ml) (Invitrogen, Milano, Italy) for 15 min at room temperature. The slides were observed with a Nikon confocal microscopy. A single composite image was obtained by superimposition of twenty optical sections for each sample observed.

### Statistical analysis

Results are expressed as the mean ± SE. Statistical comparisons between two samples were performed using the Student's t-test, whereas multiple comparisons were performed using ANOVA test as indicated; all statistical tests were performed using GraphPad Prism 6 software. In all cases, a p-value of <0.05 was considered significant.

## Results

### Culture supernatants of both classic and non-classic Th1 clones induce morphological changes of SFbs

**Fig 1:**
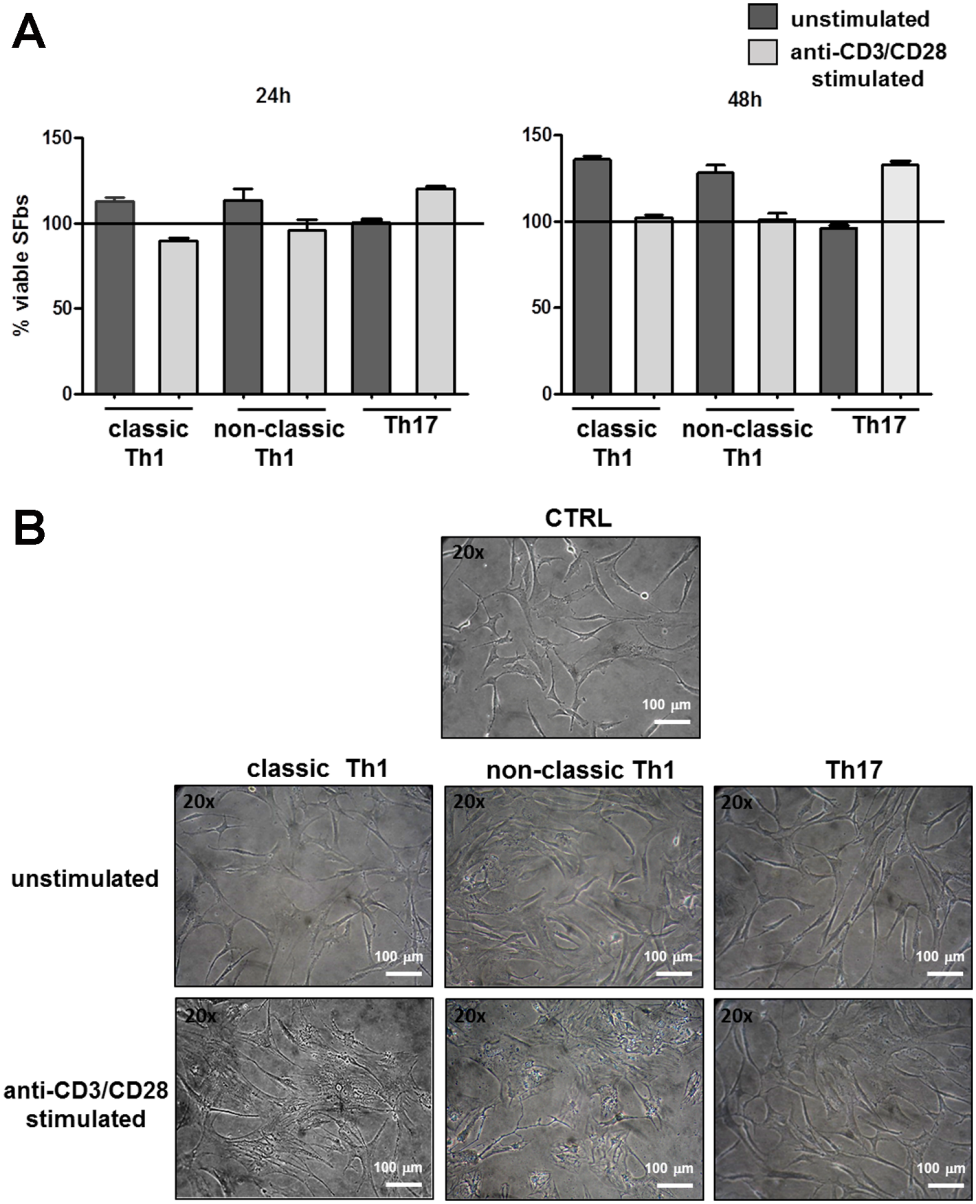
Effects of culture supernatants of Th cells clones on SFbs. SFbs from healthy donors were cultured in presence of medium (CTRL) or culture supernatants of unstimulated or anti-CD3/CD28 stimulated T helper cells of different phenotypes (classic and non-classic Th1 and Th17). **A**. SFbs vitality was evaluated by WST-1 assay after 24h and 48h of culture. Columns represent mean ± SE of % of viable SFbs compared to control condition (defined as 100% and shown as line in the Fig) in three experiments. Statistical analysis was performed by using the ANOVA test. **B**. After 48h SFbs morphology was evaluated by phase contrast microscope (magnification 200X). One representative experiment out of six is shown (one field out of five). Scale bar in all images = 100μm.

First, we evaluated the ability of culture supernatants obtained from different T helper cell subsets to influence both SFbs viability and cell shape. To this end, SFbs isolated from healthy subjects, were cultured in the presence or in the absence of supernatants obtained from both unstimulated and polyclonally- stimulated, Th17, classic and non-classic Th1 cell clones (TCCs). After 24 and 48 hours of culture, neither classic and non-classic Th1, nor Th17, supernatants, induced cell death of SFbs (Fig 1A). Interestingly, supernatants of polyclonally-stimulated Th1 cultures, both classic and non-classic, were able to induce morphological changes of SFbs compared to the unstimulated ones (Fig 1B). In particular, SFbs, which are normally fusiform and characterized by regular shape, upon 48h treatment with Th1 (classic and non-classic) supernatants, displayed a polygonal cell body, the nucleus was large and oval-shaped, and there were many slender protrusions around cell body with many branches extended out [24]. No differences were observed in presence of Th17 supernatants (Fig 1B).

### Etanercept inhibits the CD106 upregulation in SFbs selectively induced by culture supernatants of both classic and non-classic Th1 clones

**Fig 2:**
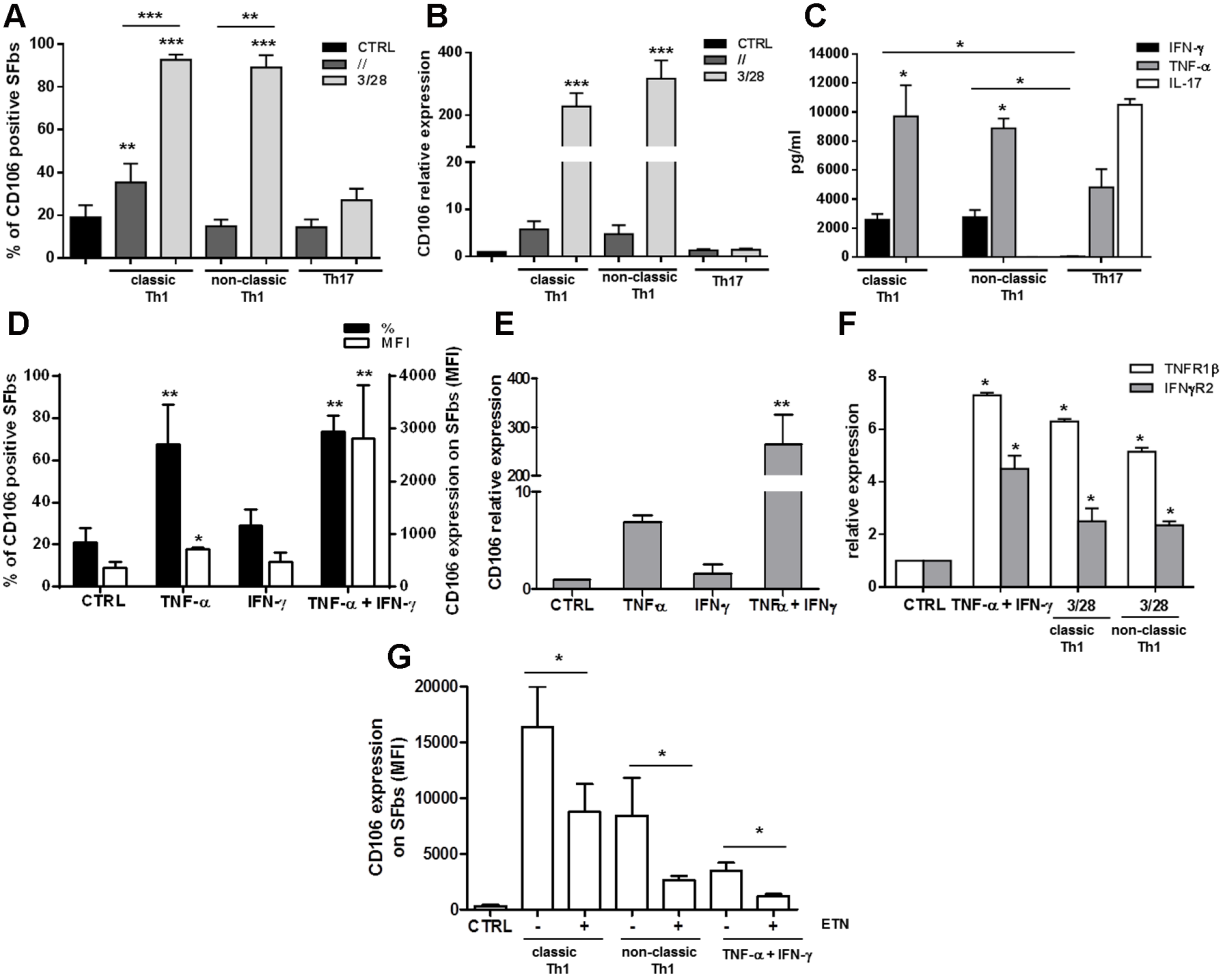
Th1-culture supernatants and TNF-α upregulate CD106 expression on SFbs. SFbs from healthy donors were cultured for 48h in presence of medium alone (CTRL) or cultured supernatants of unstimulated (dark grey) or anti-CD3/CD28 stimulated (light grey) Th cells clones of different phenotypes (classic and non-classic Th1 and Th17) (A-B) or in the presence of TNF-α or IFN-γ cytokines or their combinations (D-E) in presence or absence of Etanercept (ETN) (G). Columns represent mean ± SE of % of CD106 positive SFbs (A) or of CD106 mean fluorescence intensity (MFI) (G) or both % of CD106 positive SFbs and CD106 mean fluorescence intensity (MFI) (D) of six different experiments. After 24h CD106 (B-E), TNF-αRβ (white) and IFN-γR2 (grey) (F) expression were evaluated by real time RT-PCR. Columns represents mean ± SE of mRNA expression (normalized on housekeeping gene mRNA and calculated as fold to the control, Ctrl) of three different experiments. * p < 0.05; ** p < 0.01, *** p < 0.001 stimulated condition versus ctrl or indicated by bar. IFN-γ (black), TNF-α (grey) and IL-17 (white) cytokine levels in anti-CD3/CD28 stimulated classic and non-classic Th1 and Th17-derived supernatants were evaluated by CBA flex set assay (C); columns represents mean ± SE of cytokine concentration (pg/ml) of five different supernatants for each Th phenotype.* p < 0.05 TNF-α versus IFN-γ or indicated by bar. Statistical analysis was performed by using the ANOVA test.

Culture supernatants derived from the different subsets of TCCs were then evaluated for their ability to modulate the expression of SFbs surface markers. In particular, CD105, CD29, CD90, CD106, and CD73 expression, were evaluated, by flow cytometry, after 48 hours of culture in the presence or in the absence of supernatants obtained from both unstimulated and polyclonally-stimulated, Th17, classic and non-classic Th1 clones. As shown in Fig 2A, the frequency of CD106 (VCAM-1) positive cells resulted significantly increased in the presence of supernatants derived from both classic and non-classic Th1-, but not from Th17-, clones, whereas the frequencies of all other makers was completely unaffected (S1 Fig). These data were confirmed also in terms of CD106 mRNA expression, which was clearly increased in presence of polyclonally-stimulated Th1, both classic and non-classic, supernatants, whereas it was not affected in presence of both unstimulated and polyclonally-stimulated Th17 supernatants (Fig 2B). Evaluation of cytokines levels in anti-CD3/CD28 stimulated T cell clones supernatants, showed that both TNF-α and IFN-γ were present in Th1 (classic and non-classic)-derived supernatants and in particular that TNF-α levels were significantly higher than IFN-γ levels; moreover in Th17-derived supernatants IFN-γ resulted virtually absent and TNF-α levels were significantly lower than in Th1-derived ones (Fig 2C). As expected, IL-17 was present only in Th17- derived supernatants (Fig 2C). In order to evaluate the involvement of different cytokines in the CD106 upregulation, SFbs were cultured in presence of TNF-α, IFN-γ or their combination. Of note, none of the above described culture conditions induced apoptosis of SFbs (S2 Fig). TNF-α was able to induce a significant increase of CD106 expression at both protein and mRNA level, whereas IFN-γ alone wasn’t. More importantly, TNF-α and IFN-γ together, exerted a synergistic effect in the upregulation of CD106 expression at both protein (Fig 2D) and mRNA level (Fig 2E). In the case of flow cytometric analysis of protein, even if both cytokines did not induce a significant increase of the frequency of CD106 positive SFbs compared to TNF-α alone (Fig 2D), we found a significant increase of CD106 protein expression evaluated by mean fluorescence intensity (MFI). Since it has been previously demonstrated on different cell types a reciprocal upregulation of TNFα-RII and of IFN-γR, in response to IFN-γ and TNF-α, respectively, [25, 26], we looked at these also on SFbs. We found a synergistic effect of TNF-α and IFN-γ in the upregulation of both receptors and, more importantly, a similar upregulation was observed in SFbs cultured in presence of Th1 classic and non-classic–derived supernatants (Fig 2F). TNF-α is a pro-inflammatory cytokine that plays crucial role in the pathogenesis of several forms of arthritis, and, as a consequence, it represents a therapeutic target in such diseases. For these reasons, we decided to assess if etanercept (ETN), a soluble dimeric fusion protein of p75 TNFR that belongs to the class of TNF-inhibitors, was able to interfere also with SFbs CD106 upregulation mediated by Th1-derived supernatants. To this aim SFbs were cultured for 48h in presence of polyclonally stimulated Th1 classic and non-classic derived supernatants, or in presence of exogenous IFN-γ and TNF-α, with or without ETN. We observed a significant reduction of CD106 mean fluorescence intensity in presence of ETN in all the culture conditions (Fig 2G).

### Leukocytes adhesion to SFbs is mediated by CD106 expression

**Fig 3:**
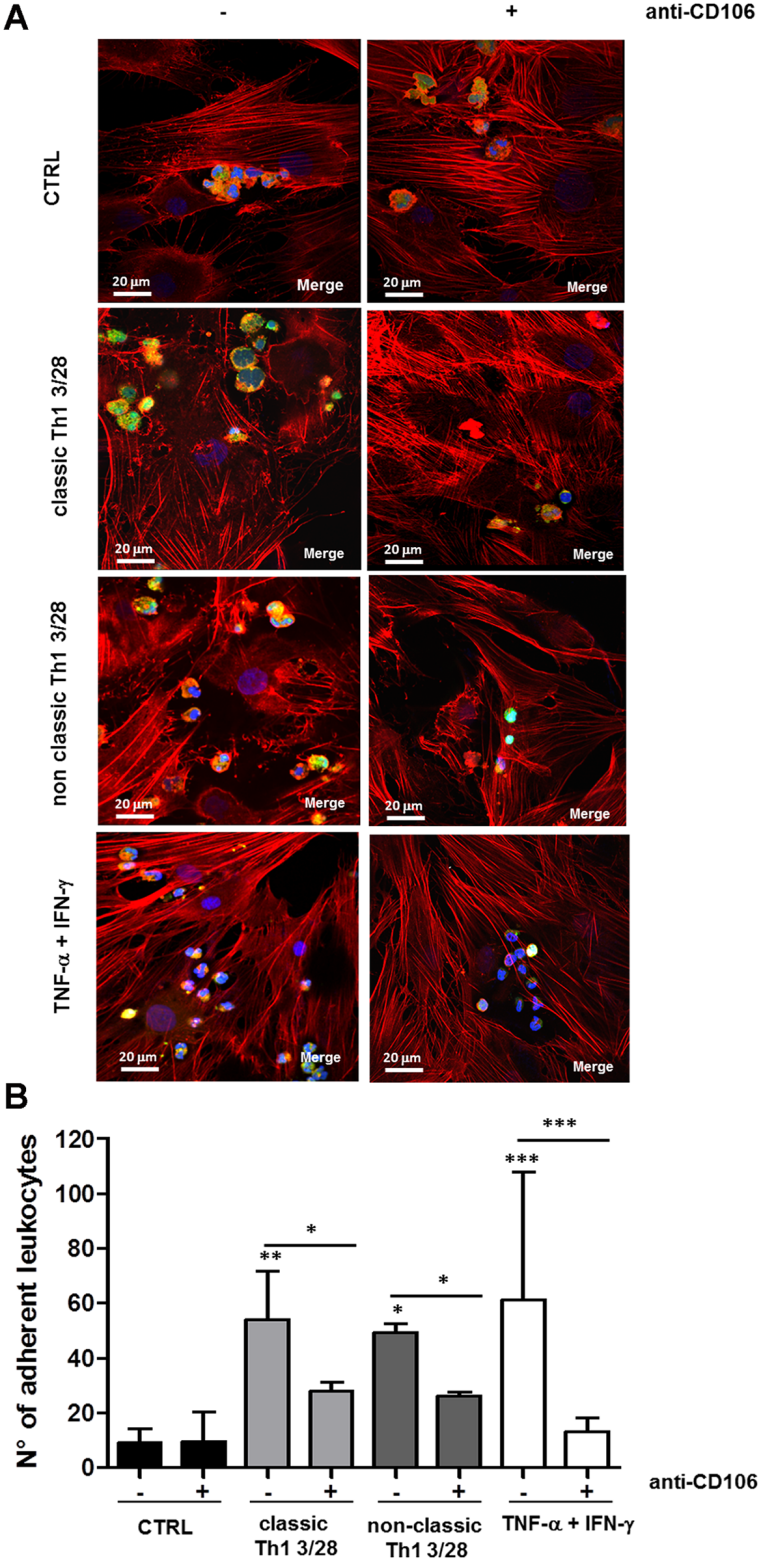
CD106 upregulation induced by Th1 clones, mediates leukocytes adhesion on SFbs. SFbs from healthy donors were cultured for 48h in presence of medium alone (CTRL), supernatants of anti-CD3/CD28 stimulated classic and non-classic Th1 cells clones, or TNF-α plus IFN-γ; then CFSE-labelled leukocytes derived from peripheral blood of healthy donors were cultured for 2h on treated SFbs, in presence or absence of anti-CD106 neutralizing mAb. Leucocytes adhesion on SFbs was observed by confocal microscopy analysis (**A**, magnification 400x, scale bar in all images = 20 μm; one representative experiment out of three and of this one field out of five is shown); or by fluorescence microscope analysis by average of adherent leucocytes counted in five different random fields (**B**, columns represent mean ± SE of number of adherent leucocytes of three different experiments). * p < 0.05; ** p < 0.01, *** p < 0.001 stimulated condition versus ctrl or indicated by bar. Statistical analysis was performed by using the ANOVA test.

CD106 is a sialoglycoprotein expressed by cytokine-activated endothelium which mediates leukocyte-endothelial cell adhesion and signal transduction. In order to check the biologic relevance of the TNF-α induced CD106 upregulation on SFbs, we performed cell adhesion experiments in which the numbers of leukocytes adhering to SFbs have been evaluated in different experimental conditions. Treatment of SFbs with TNF-*α* plus IFN-γ, as well as with polyclonally-stimulated, classic and non-classic Th1 supernatants, resulted in an increased ability of leukocytes to adhere to SFbs as compared to the untreated SFbs (Fig 3A and 3B). More importantly, the addition in culture of a neutralizing anti-CD106 mAb, significantly reduced leukocyte adhesion in all culture conditions (Fig 3B), this effect being not exerted by an isotype control Ab (S3A Fig). In any case, the frequencies of cell subsets among adherent leukocytes was similar in the different culture conditions (S3B Fig), suggesting that the CD106-mediated adhesion was not limited to a specific subset of leukocytes.

### CD106 is over-expressed in SFbs derived from JIA patients

**Fig 4:**
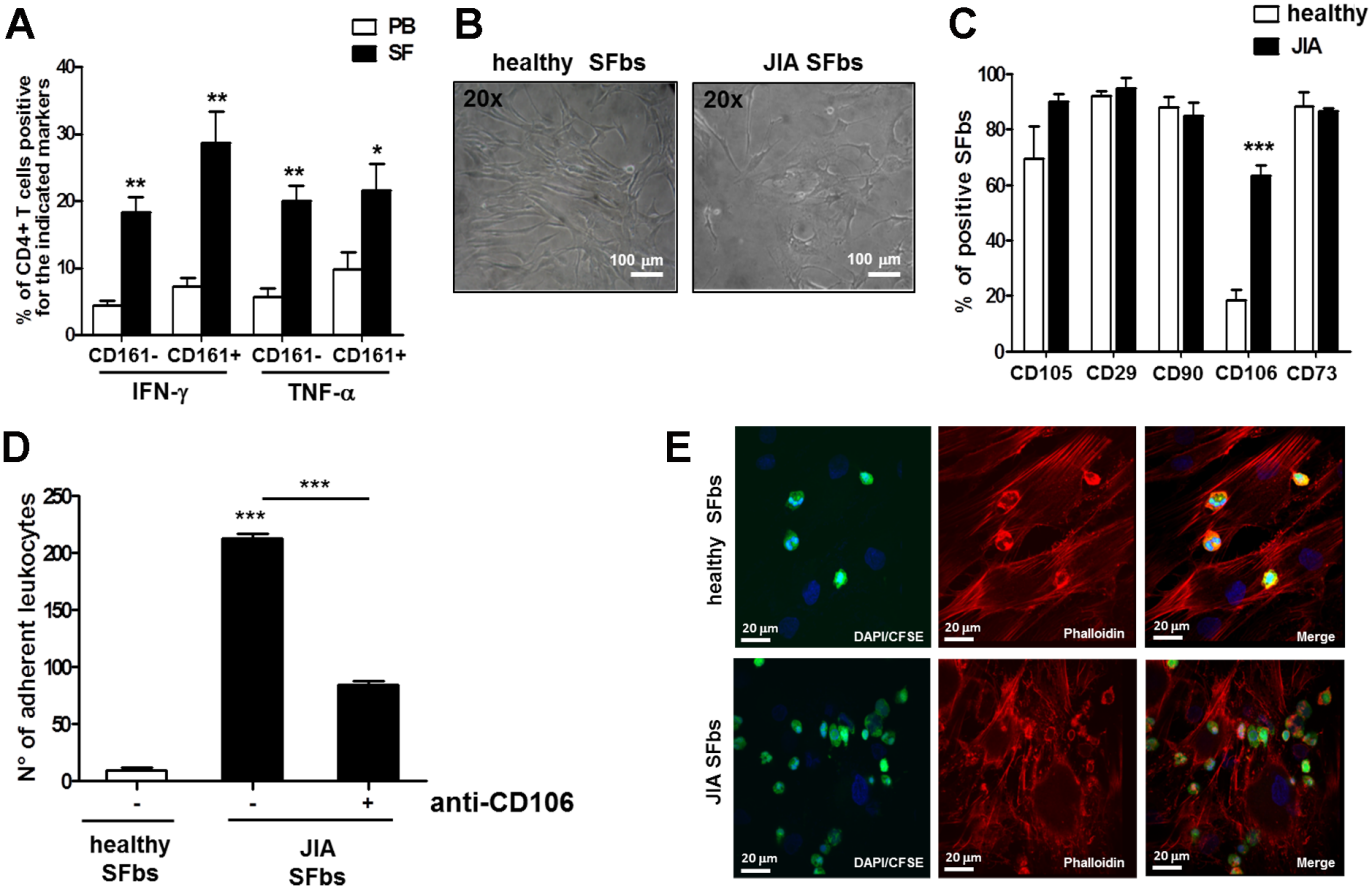
SFbs derived from synovial fluid of JIA patients express higher level of CD106 than normal SFbs. **A**. MNCs from PB and SF of JIA patients were polyclonally stimulated with PMA plus ionomycin for 6h the last 4h in presence of BFA and then evaluated for intracellular cytokines production. Columns represent mean ± SE of % of CD3+CD4+ T cells of seven JIA patients producing the indicated cytokine and expressing CD161. * p < 0.05; ** p < 0.01, *** p < 0.001 PB versus SF; **B** SFbs obtained from healthy subjects and from SF of JIA patients were evaluated by phase contrast microscope (magnification 200X, one representative experiment out of seven and of this one field out of five is shown, scale bar = 100 μm and **(C)** were characterized by flow cytometry for surface expression of CD106, CD29, CD90, CD106, CD73. Columns represent the mean ± SE of % of SFbs from six healthy donors and from seven JIA patients positive for the indicated markers. *** p < 0.001 JIA versus healthy SFbs; **D-E** CFSE-labelled leukocytes derived from PB of healthy donors were cultured for 2h on healthy- or JIA-derived SFbs, in presence or absence of anti-CD106 neutralizing mAb. Leucocytes adhesion on SFbs was evaluated by fluorescence microscope analysis by average of adherent leucocytes counted in five different random fields (**D**, columns represent mean ± SE of number of adherent leukocytes of three different experiments, *** p < 0.001 stimulated condition versus ctrl or indicated by bar) and by confocal microscopy analysis (**E**, magnification 400x, one representative experiment out of three and of this one field out of five is shown). Statistical analysis was performed by using the Student t-test (two groups) or the ANOVA test (several groups).

Once established that both classic and non-classic Th1 cells are able to induce CD106 upregulation on SFbs via TNF-α, favouring leukocyte adhesion, we moved to assess this pathway in JIA. First of all, we checked the frequencies of the different T cell subsets in the PB and in the SF of JIA patients. As expected and previously described (18), higher frequencies of IFN-γ producing cells (both CD161+ and CD161-), were found in SF than in PB of the JIA patients (Fig 4A), and more importantly we also found a significant increase of TNF-α producing cells (both CD161+ and CD161-), in SF compared to PB (Fig 4A). Moreover, SFbs isolated from SF of JIA patients showed peculiar morphological characteristics which were different from that of SFbs derived from healthy subjects and resembled those of healthy SFbs treated in vitro with classic or non-classic Th1 supernatants (Figs 4B and 1B). More importantly, only the expression of CD106, among several surface markers analysed, was significantly higher in JIA patients- than in healthy subjects-derived SFbs (Fig 4C). Accordingly, cell adhesion experiments showed significant increased ability of JIA-derived SFbs to promote spontaneous leukocytes adhesion than healthy subject-derived SFbs (Fig 4D and 4E). In addition, cell adhesion was significantly inhibited by the presence in culture of a neutralizing anti-CD106 mAb (Fig 4D), but not of an isotype control Ab (data not shown). Moreover the frequencies of different subsets of leukocytes adherent to JIA-derived SFbs were not different form that adherent to healthy SFbs (S3C Fig).

## Discussion

In inflamed joints, CD4+ T cells produce cytokines that sustain the process of synovial pannus-driven extracellular proteolysis and proliferation, and promote the inflammation-associated angiogenesis [27, 21]. The present study aimed to identify factors which may mediate the interplay between CD4+ T effector cells SFbs in terms of synovial activation and involvement of inflammatory cells in immune mediated arthritis. Since T helper lymphocytes orchestrate the inflammation mainly through the production of cytokines that act on resident and inflammatory cells present in the joints, we initially evaluated the ability of Th17, classic and non-classic Th1 clones-derived supernatants to induce adhesion molecules expression on SFbs isolated from the synovia of healthy subjects. We decided to study the above mentioned CD4+ T cell subsets because we have previously shown that IL-12, present in SF of JIA patients, induces Th17 cells to produce IFN-γ thus explaining the high frequency of non-classic Th1 cells in the inflammatory environment [28]. In the present study we demonstrated that both classic and non-classic Th1-, but not Th17-derived supernatants, are the most powerful activators of synovial fibroblasts leading to the upregulation of CD106 expression, that is crucial for leukocytes adhesion, and consequently for their retention into the synovia. The upregulation of CD106, at both mRNA and protein level, was induced by TNF-α alone, whereas IFN-γ, that had no effects when administered alone, significantly synergized with TNF-α in the induction of CD106 expression. This synergistic effect could be explained by the reciprocal upregulation of TNF-αRII and IFN-γR, in response to IFN-γ and TNF-α, respectively, as previously demonstrated in several cell types [25, 26] and confirmed also in SFbs in the present study. Indeed, both the cocktail containing TNF-α and IFN-γ and the supernatants derived from classic and non-classic Th1 cells, induced upregulation of TNF-αRII and IFN-γRII mRNA. Moreover, evaluation of cytokine level in Th derived supernatants, explains the different behaviour of Th17 and Th1 (classic and non-classic) in the regulation of CD106 expression and supports the main role of TNF-α and IFN-γ. An additional important finding of this study is that, in vitro administration of the TNF-α inhibitor etanercept, was able to significantly reduce the CD106 expression induced by both classic and non-classic Th1 supernatants. Of note, TNF-α-blocking agents, such as etanercept, induce amelioration of clinical symptoms, laboratory parameters of inflammation, and radiological progression in patients with immune-mediated arthritis, such as RA, JIA, and psoriatic arthritis [29]. The pleiotropic activity of TNF-α in the inflammatory milieu includes activation of monocytes, macrophages and synovial fibroblasts by inducing the transcription of genes whose products mediate inflammation and tissue degradation, leading to cartilage and bone destruction. Moreover, TNF-α induces the growth of the synovial membrane with neovascularization and the formation of the 'pannus', which contains osteoclasts. TNF-α can also act on T lymphocytes, by driving the shifting from Th17 to non-classic Th1 cells [30]. The upregulation of CD106 expression on SFbs may represent a further pro-inflammatory effect of TNF-α, allowing the retention of leukocytes into the synovia and contributing to amplify the inflammation. The finding that CD106 expression is significantly higher on SFbs from JIA patients compared to healthy donors, supports this hypothesis. In addition, the morphology of JIA SFbs resembled the one obtained in vitro by activating healthy subjects-derived SFbs with classic and non-classic Th1 supernatants, suggesting that in vivo, the TNF-α rich microenvironment of inflamed joints of JIA patients, may favour a cytoskeleton reorganization leading to morphological changes and ultimately resulting in “transformed phenotype” of these cells [24].

Another important information emerging from this study, is about the crucial role of CD106 in leukocytes adhesion to SFbs. The fact that adhesion molecules present on the SFbs surface regulate the trafficking of leukocytes into and/or through the synovial tissues has been reported previously [31]. The increase in CD106 expression and associated endothelial activation contributes to the promotion of inflammation and tissue damage in several different autoimmune diseases, such as rheumatoid arthritis (RA), systemic lupus erythematous (SLE) and scleroderma [32]. Here we found that in presence of neutralizing anti-CD106 mAb, the TNF-α-induced adhesion of leucocytes to SFbs, was completely blocked, and the adhesion induced by Th1 derived supernatants was significantly reduced. The result of these experiments strongly suggest that CD106 is, among adhesion molecules, the most important for leukocyte adhesion to SFbs. This concept is further strengthened by the results obtained in SFbs from JIA patients, that show a spontaneous high expression of CD106, and a high rate of leukocyte adhesion, confirming the crucial role of TNF-α/CD106 axis in leukocyte adhesion to SFbs [33].

Even if previous studies have been reported that TNF-α upregulates VCAM-1 and ICAM-1 in RA-derived SFbs [34, 35], to our knowledge this is the first study aimed to characterize the interplay between different T cell subsets and JIA-derived SFbs. In particular the results obtained both in the in vitro model of the healthy-derived Th effector clones of different phenotypes and in the ex vivo models of JIA, concordantly indicate that Th1 cells, both classic and non-classic, induced CD106 expression on SFbs through TNF-α, allowing leukocytes adhesion. The understanding of the possible cellular interaction arising in the inflamed joints of RA and JIA patients and the identification of new molecular pathways in joint inflammatory disorders, could represent an important issue to develop new therapeutic strategies in such diseases.

## Supporting Information

S1 Fig Phenotypical analysis of SFbs in presence of culture supernatants of Th cell clones. SFbs from healthy donors were cultured in presence of medium (CTRL) or cultured supernatants of unstimulated or anti-CD3/CD28 stimulated Th cell clones of different phenotypes (classic and non-classic Th1 and Th17). After 48h, SFbs were evaluated by flow cytometry for the indicated surface markers. Columns represent mean ± SE of % of positive SFbs of six different experiments. Statistical analysis was performed by using the ANOVA test.
(TIF)

S2 Fig Effects of TNF-α and IFN-γ on SFbs viability. SFbs from healthy donors were cultured in presence of medium alone or TNF-α or IFN-γ or their combination. SFbs vitality was evaluated by WST-1 assay after 24h and 48h of culture. Columns represent mean ± SE of % of viable SFbs compared to control condition (defined as 100%) in three experiments. Statistical analysis was performed by using the ANOVA test.
(TIF)

S3 Fig Adhesion to SFbs is similar for all leukocyte subsets. SFbs from healthy donors were cultured for 48h in presence of medium alone (CTRL) or supernatants of anti-CD3/CD28 stimulated classic (A and B) and non-classic Th1 (B) cells clones or TNF-α plus IFN-γ (A and B) and in presence of anti-CD106 mAb (A and B) or isotype control (A); then CFSE-labelled leukocytes derived from PB of healthy donors were cultured for 2h on treated SFbs. Leucocytes adhesion on SFbs was evaluated by fluorescence microscope analysis by average of adherent leucocytes counted in five different random fields (**A**, columns represent mean ± SE of number of adherent leukocytes of three different experiments, ** p < 0.01, *** p < 0.001 stimulated condition versus ctrl or indicated by bar). Leukocytes recovered after adhesion assay were analysed by flow cytometry to identify the main cell subsets (neutrophils CD15+, monocytes CD14+, T cells CD3+, B cells CD19+). **B** Columns represent mean ± SE of the frequency of each population of leukocytes adherent to SFbs in three different experiments. Statistical analysis was performed by using the ANOVA test. **C)** Leukocytes derived from PB of healthy donors were cultured for 2h on JIA-derived SFbs, Leukocytes recovered after adhesion assay were analysed by flow cytometry to identify the main cell subsets (neutrophils CD15+, monocytes CD14+, T cells CD3+, B cells CD19+). Columns represent mean ± SE of % of cells of each population of leukocytes adherent to SFbs in four different experiments.
(TIF)
