# Supplementary figures and images for: Th1-Induced CD106 Expression Mediates Leukocytes Adhesion on Synovial Fibroblasts from Juvenile Idiopathic Arthritis Patients
Source: PLoS One. 2016 Apr 28;11(4):e0154422. doi: 10.1371/journal.pone.0154422 (PMC4849574; doi:10.1371/journal.pone.0154422)

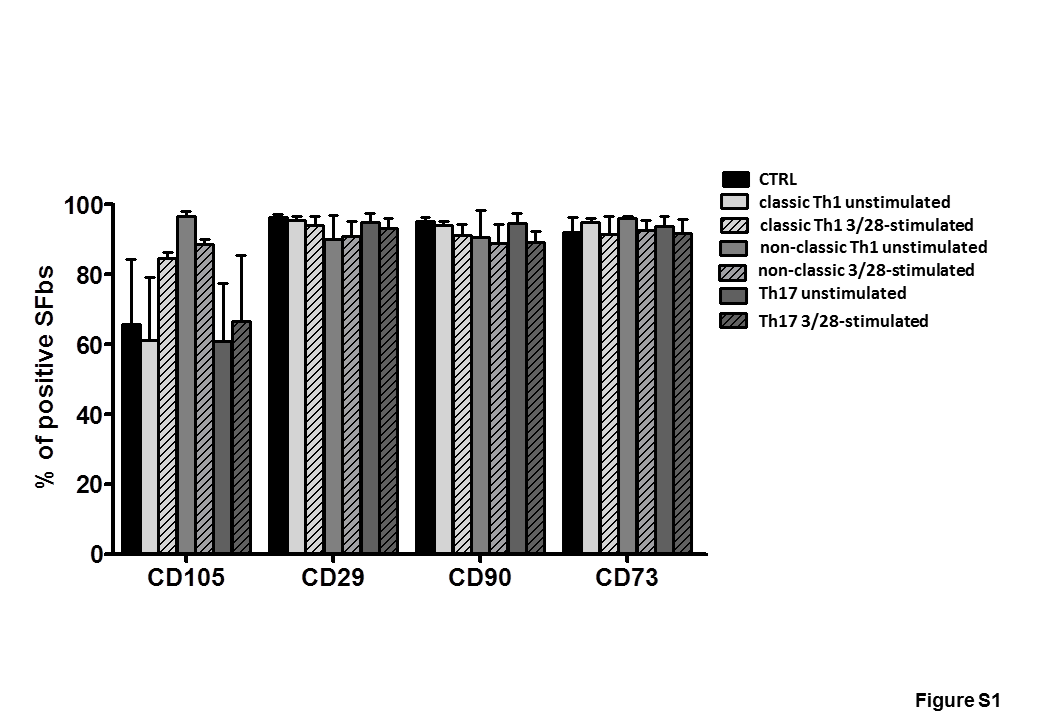

Supplement: S1 Fig — SFbs from healthy donors were cultured in presence of medium (CTRL) or cultured supernatants of unstimulated or anti-CD3/CD28 stimulated Th cell clones of different phenotypes (classic and non-classic Th1 and Th17). After 48h, SFbs were evaluated by flow cytometry for the indicated surface markers. Columns represent mean ± SE of % of positive SFbs of six different experiments. Statistical analysis was performed by using the ANOVA test. (TIF) [file pone.0154422.s001.tif]

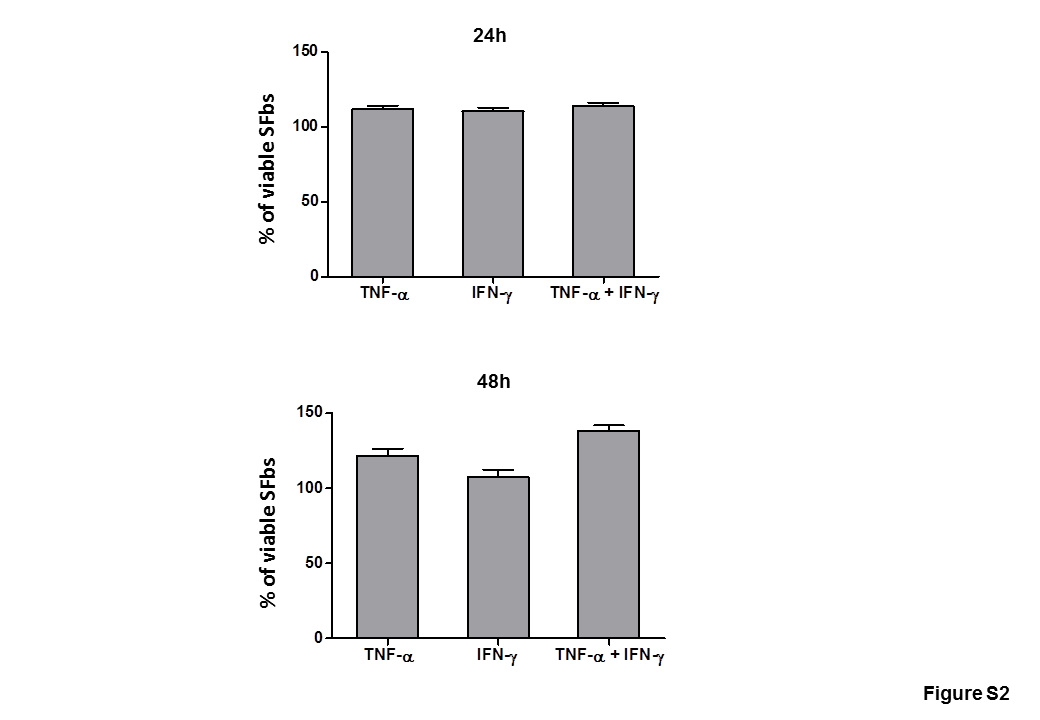

Supplement: S2 Fig — SFbs from healthy donors were cultured in presence of medium alone or TNF-α or IFN-γ or their combination. SFbs vitality was evaluated by WST-1 assay after 24h and 48h of culture. Columns represent mean ± SE of % of viable SFbs compared to control condition (defined as 100%) in three experiments. Statistical analysis was performed by using the ANOVA test. (TIF) [file pone.0154422.s002.tif]

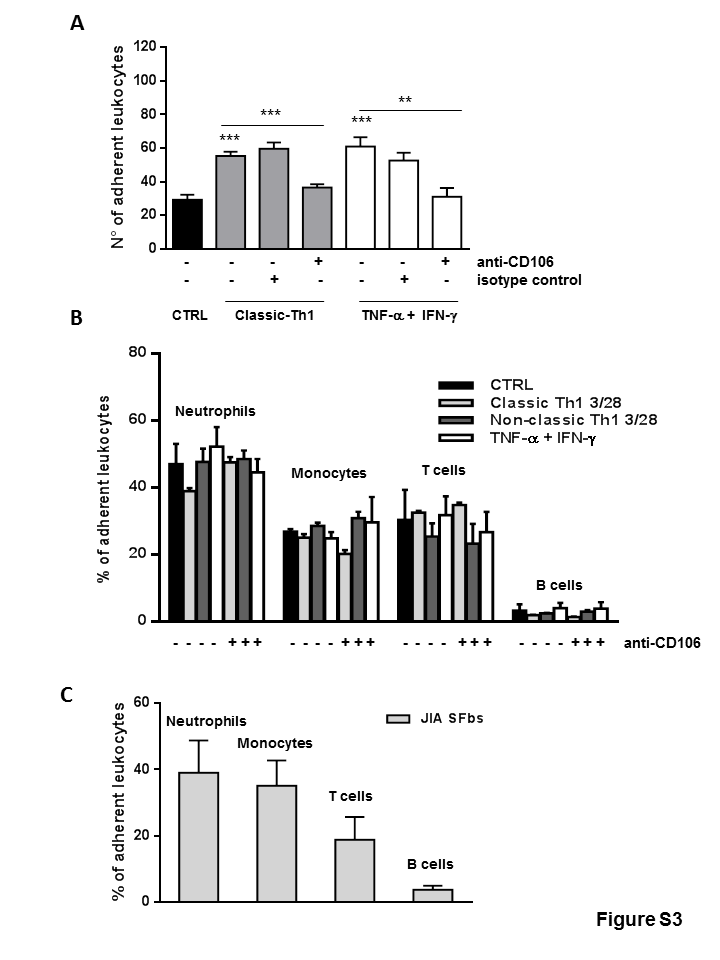

Supplement: S3 Fig — SFbs from healthy donors were cultured for 48h in presence of medium alone (CTRL) or supernatants of anti-CD3/CD28 stimulated classic (A and B) and non-classic Th1 (B) cells clones or TNF-α plus IFN-γ (A and B) and in presence of anti-CD106 mAb (A and B) or isotype control (A); then CFSE-labelled leukocytes derived from PB of healthy donors were cultured for 2h on treated SFbs. Leucocytes adhesion on SFbs was evaluated by fluorescence microscope analysis by average of adherent leucocytes counted in five different random fields (A, columns represent mean ± SE of number of adherent leukocytes of three different experiments, ** p < 0.01, *** p < 0.001 stimulated condition versus ctrl or indicated by bar). Leukocytes recovered after adhesion assay were analysed by flow cytometry to identify the main cell subsets (neutrophils CD15+, monocytes CD14+, T cells CD3+, B cells CD19+). B Columns represent mean ± SE of the frequency of each population of leukocytes adherent to SFbs in three different experiments. Statistical analysis was performed by using the ANOVA test. C) Leukocytes derived from PB of healthy donors were cultured for 2h on JIA-derived SFbs, Leukocytes recovered after adhesion assay were analysed by flow cytometry to identify the main cell subsets (neutrophils CD15+, monocytes CD14+, T cells CD3+, B cells CD19+). Columns represent mean ± SE of % of cells of each population of leukocytes adherent to SFbs in four different experiments. (TIF) [file pone.0154422.s003.tif]
